# DEVELOPMENT OF A TRAINING MODEL WITHIN HOSPICE AND PALLIATIVE CARE FOR NURSE PRACTITIONERS

**DOI:** 10.1093/geroni/igae098.3101

**Published:** 2024-12-31

**Authors:** Alex Laffer, Chandrika Kumar, Tracy Shamas, Sarah Gillespie-Heyman, Alexandra Podosek, Andrea Ruskin

**Affiliations:** VA Connecticut Healthcare System, New Haven, Connecticut, United States; VA Connecticut Healthcare System, West Haven, Connecticut, United States; VA Connecticut Healthcare System, West Haven, Connecticut, United States; VA Connecticut Healthcare System, West Haven, Connecticut, United States; VA Connecticut Healthcare System, West Haven, Connecticut, United States; VA Connecticut Healthcare System, West Haven, Connecticut, United States

## Abstract

Workforce shortages within palliative care restricts availability of this service. Although clinicians may develop an interest in this specialty later in their careers, acquiring the necessary education and training is challenging. In academic medical centers like the Veteran’s Administration (VA), interprofessional palliative care teams are well-positioned to deliver high-quality, accessible palliative care services when patient needs surpass what can be addressed by primary care and surgical subspecialities. Given workforce shortages, this quality improvement project aims to increase competency for nurse practitioners (NPs) in hospice and palliative care. A 12-month, minimum 500 clinical hour, training program designed to provide clinical and skill-based education to NPs currently working on the VA Community Living Center was developed. The predominantly practical curriculum focuses on interprofessional teamwork, communication skills, and application of medical, psychosocial, cultural, spiritual, and ethical content. The curriculum is designed in a longitudinal manner that supports the NP’s ability to maintain their routine clinical care responsibilities. The program includes two hours of protected time each day consisting of hospice and palliative care clinical experiences alongside interprofessional learning. The training cohort, beginning March 2024, consists of two NPs. The team developed a mixed methods approach to assess competencies and progress across the training period. This project has resulted in an innovative approach to enhance hospice and palliative care training that will advance mid-career clinicians’ skills and help address workforce shortages without requiring clinicians to leave their jobs for extended training. Implementation will have implications for the feasibility, sustainability, and replication of this model.

